# RETRACTION: A Perspective on the Emergence of Sialic Acids as Potent Determinants Affecting *Leishmania* Biology

**DOI:** 10.1155/mbi/9897434

**Published:** 2026-03-30

**Authors:** Molecular Biology International

RETRACTION: A. Ghoshal and C. Mandal, “A Perspective on the Emergence of Sialic Acids as Potent Determinants Affecting *Leishmania* Biology,” *Molecular Biology International* 2011, no. 1 (2011): 532106, https://doi.org/10.4061/2011/532106.

The above article, published online on 25 July 2011 in Wiley Online Library (https://wileyonlinelibrary.com), has been retracted by John Wiley & Sons Ltd. The retraction has been agreed after an investigation into figure concerns raised by *Melanocorypha yeltoniensis* and *Gigantochloa parviflora* via PubPeer [1].

Specifically, the following concerns were noted:

- Unexpected similarities among several of the lanes in Figure 2(b);
- Unexpected similarities among the lanes in Figure 3(d);
- Unexpected similarities between lanes in Figure 3(d) and Figure 3C of another publication by some of the same authors [2];
- Unexpected similarities between the flow cytometry data in Figure 4(d) and 4(e) with Figure 5C of another publication by some of the same authors [3].

Further investigation also identified the following concerns:

- Unexpected similarities between the fluorescence micrographs in Figure 4(c) with Figure 4 in [3].

Separate attempts by the journal to contact the authors have not resulted in a reply. As such, the results and conclusions of this article are considered to be unreliable, and therefore the article must be retracted.
